# Cerebroprotective Effects of Dimeric Copper(II) Bis(o-acetoxybenzoate) on Ischemia-reperfusion Injury in Gerbils

**DOI:** 10.1155/MBD.2002.253

**Published:** 2002

**Authors:** Ling Li, Zhiqiang Shen, Weimin Yang, Wanling Wu, Weiping Liu, Zhihe Chen

**Affiliations:** 1 Yunnan Pharmacological Laboratories of Natural Products Kunming Medical College Kunming 650031 China; 2 Kunming Institute of Precious Metals Kunming 650221 China

## Abstract

The cerebroprotective effects of copper aspirinate [dimeric copper(II) bis(o-acetoxybenzoate)] were investigated in gerbils subjected to 10-min global cerebral ischemia followed b 60-min reperfusion. The results showed that intragastric copper aspirinate (7.5, 15.0 and 30.0 mg Kg^−1^) markedly promoted the recovery of the electroencephalogram amplitude, attenuated the increase of lipid peroxide content and the decrease of superoxide dismutase activity in the cortex during ischemia-reperfusion injury. It suggested that copper aspirinate possesses potential neuroprotective properties, the mechanism of which might be related to an increase of the activity of endogenous superoxide dismutase.


### CEREBROPROTECTIVE EFFECTS OF DIMERIC COPPER(II) BIS(o-ACETOXYBENZOATE) ON ISCHEMIA-REPERFUSION INJURY IN GERBILS

Ling Li*[1], Zhiqiang Shen[1], Weimin Yang[1], Wanling Wu[1], Weiping Liu[2], and Zhihe Chen[1]

[1] Yunnan Pharmacological Laboratories of Natural Products, Kunming Medical College, Kunming 650031 < kmliling@hotmail.com>
[2] Kunming Institute of Precious Metals, Kunming 650221, China

### ABSTRACT

The cerebroprotective effects of copper aspirinate [dimeric copper(II) bis(o-acetoxybenzoate)] were investigated in gerbils subjected to 10-min global cerebral ischemia followed by 60-min reperfusion. The results showed that intragastric copper aspirinate (7.5, 15.0 and 30.0 mg $kg^{-1}$) markedly promoted the recovery of the electroencephalogram amplitude, attenuated the increase of lipid peroxide content and the decrease of superoxide dismutase activity in the cortex during ischemia-reperfusion injury. It suggested that copper aspirinate possesses potential neuroprotective properties, the mechanism of which might be related to an increase of the activity of endogenous superoxide dismutase.
**Key words** copper aspirinate, cerebral ischemia-reperfusion, electroencephalogram, antioxidant enzyme, lipid peroxide

### INTRODUCTION

Copper aspirinate [dimeric copper (II) bis(o-acetoxybenzoate)], a copper salt of aspirin (Fig. 1), has been demonstrated to exhibit more potent anti-inflammation and antiplatelet activities than aspirin [1,2], as well as lower gastrointestinal side effects than aspirin [3]. These facts suggest that copper aspirinate might be more advantageous in preventing and treating ischaemic stroke. In a previous study, the protective effects of copper aspirinate on cerebral ischemia-reperfusion injury were evidenced by a decrease of the gerbil mortality, increase of the intact neural density in the posterior CA1 sector of hippocampus and attenuation of the cortex calcium accumulation in cerebral ischemia-reperfusion gerbils [4]. In the present study, the neuroprotective effects of copper aspirinate are further investigated by determining the electroencephalogram (EEG), lipid peroxide (LPO) and antioxidant enzymatic activities in gerbils subjected to cerebral ischemia-reperfusion injury.

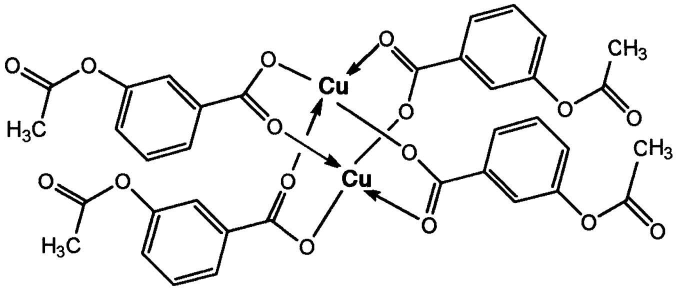

**Fig.** Structure of the copper aspirinate

### MATERIALS AND METHODS

### *Chemicals and Drugs*

Copper aspirinate (Cu 14.99 %, C 51.21 %, H 3.32 %; purity > 98%) was synthesized by the Kunming Institute of Precious Metals. It was dissolved in 0.9 % saline (pH 6.5). Crystalline aspirin was dissolved in 100 mmol $L^{-1}$ $Na_2CO_3$. Kits for determining the activities of antioxidant enzymes (superoxide dismutase, SOD; glutathione peroxidase, GSH-PX) were purchased from the Nanjing Jianchen Institute of Biotechnology. 1,1,3,3-Tetramethoxypropane was from Fluka. Other reagents were of analytical grade.

### *Animals*

The adult gerbils of either sex, weighting 50~70 g (obtained from the Yunnan Pharmacological Laboratories of Natural Products) were used in this study in accordance with the ethics committee of our Laboratories.

### *Induction of cerebral ischemia-reperfusion*

The gerbils were randomly divided into 6 groups with 6 gerbils each. Group A (sham-operation, sham) and group B (ischemia-reperfusion, Isc-Rep): the same volume of saline (20 ml $kg^{-1}$), groups C-E: 7.5,

15.0 and 30.0 mg $kg^{-1}$ copper aspirinate and group F: 30 mg $kg^{-1}$ aspirin. All the above substances were administered intragastrically twice daily for five dosages prior to ischemia. An hour after administration of the fifth dosage, the model of cerebral ischemia-reperfusion injury was prepared by occlusion of the bilateral common carotid arteries with atraumatic arterial clips for 10 min followed by 60 min reperfusion under anesthetizing with sodium pentobarbital (50 mg $kg^{-1}$). In sham-operated gerbils the arteries were exposed but not occluded. Body temperature of animals was maintained at 36.5~37.5 °C during anesthesia with a heater. At the end of experiment, the gerbils were killed and the forebrain cortex was removed quickly for the determination of LPO content, antioxidant enzymatic activities.

***EEG recording and analyses***

A silver electrode connected to a memory oscilloscope (VC-11, Nihon Kohden) was inserted subcutaneously in the front parietal region. The reference electrode was placed at the region of the occiput. EEG was obtained on an X-Y recorder (RW-21S, Rikendeki) before ischemia and at 0, 10, 30, 60 min after reperfusion and examined by an Averager / Histogram Analyzer (QC-111J, Nihon Konden). Percentage of EEG amplitude was calculated according to the formula:

EEG amplitude (%) = A or B / C x 100

where A, B and C are EEG amplitude during ischemia, reperfusion, and before ischemia, respectively.

***Measurement of antioxidant enzymatic activities***

At the end of the experiment, the forebrain cortex was removed quickly and stored at –30 °C. On the day of the assay, each cortex was defrosted and homogenized. The homogenate was centrifuged at 4000 rev $min^{-1}$ for 15 min at 4 °C to obtain a supernatant. The antioxidant enzymatic activities in the supernatant were measured using commercially available kits. The protein in the supernatant was measured as described by Bradford [5].

***Measurement of LPO contents***

The content of LPO in the supernatant was measured with thiobarbituric acid as described previously [6]. The level of LPO was expressed as nmol $mg^{-1}$ protein of malondialdehyde (MDA) using 1,1,3,3,-tetramethoxypropane as external standard.

***Statistics***

Data were expressed as means ± s.d. Statistical significance of differences between groups was determined by Student's *t*-test.

### RESULTS AND DISCUSSION

After occlusion of the bilateral common carotid arteries, the amplitude of EEG was immediately reduced and even became flattened in the Isc-Rep group. During reperfusion, the EEG amplitude recovered slowly and reached only 32.4 % at 60 min after reperfusion. Intragastric administration of aspirin 30.0 mg $kg^{-1}$ markedly promoted the recovery of the EEG amplitude to 58.4 %. The evident recovery of the EEG amplitude was also observed in the copper aspirinate-treated groups (7.5, 15.0, and 30.0 mg $kg^{-1}$), with recoveres of 60.9, 68.5 and 70.4 % at 60 min after reperfusion, respectively ($P$ < 0.05, 0.01 compared with the Isc-Rep group). (Table I)

**Table I. Effect of copper aspirinate administered intragastrically on EEG during cerebral ischemia / reperfusion in gerbils**

| Groups | Dose ($mg\ kg^{-1}$) | EEG amplitude (%) Ischemia 10 min | Reperfusion 10 | 30 | 60 (min) |
|---|---|---|---|---|---|
| Sham | Saline | — | 101.5 ± 10.9$^{**}$ | 105.9 ± 9.2$^{**}$ | 105.5 ± 9.6$^{**}$ |
| Isc-Rep | Saline | 17.9 ± 7.2 | 21.5 ± 4.5 | 23.4 ± 3.6 | 32.4 ± 5.2 |
| Copper aspirinate | 7.5 | 20.6 ± 6.1 | 30.7 ± 7.7$^{*}$ | 44.9 ± 7.3$^{**}$ | 60.9 ± 6.5$^{**}$ |
| | 15.0 | 20.6 ± 4.4 | 31.0 ± 7.1$^{*}$ | 48.8 ± 9.8$^{**}$ | 68.5 ± 10.9$^{**}$ |
| | 30.0 | 21.4 ± 5.3 | 34.4 ± 11.0$^{*}$ | 52.3 ± 7.3$^{**}$ | 70.4 ± 4.0$^{**}$ |
| Aspirin | 30.0 | 20.5 ± 5.7 | 37.5 ± 5.4$^{**}$ | 49.6 ± 11.3$^{**}$ | 58.4 ± 9.7$^{**}$ |

Cerebral ischemia-reperfusion injury was produced by occlusion of the bilateral common carotid arteries for 10 min followed by 60 min reperfusion in gerbils. Drugs were administered intragastrically twice daily for five dosages prior to ischemia. Values were means ± s.d, $n$ = 6. $^{*}P$ <0.05, $^{**}P$ <0.01 compared with Isc-

Rep group by Student's *t*-test.

Our previous study confirmed that copper aspirinate possesses cerebroprotective effect in morphology [4]. In this study, copper aspirinate significantly promoted the recovery of the EEG amplitude during reperfusion, further demonstrating its neuroprotection in electrophysiology. It was worth mentioning that copper aspirinate given intragastrically at 7.5 mg $kg^{-1}$ produced a nearly equal efficacy to aspirin 30 mg $kg^{-1}$, which might be related to its increasing the plasma level of prostacyclin [7]. Prostacyclin is known as a powerful vasodilator. Recently, copper aspirinate was reported to relax vascular smooth muscles [8] and inhibit platelet-neutrophil adhesion [9]. These pharmacological actions would contribute to improving brain circulation.

**Table II. Effects of intragastric copper aspirinate on MDA content and antioxidant enzymatic activities of cortex after 60 min reperfusion in gerbils**

| Groups | Dose mg $kg^{-1}$ | MDA (nmol $mg^{-1}$ protein) | SOD (NU $mg^{-1}$ protein) | GSH-PX (U $mg^{-1}$ protein) |
|---|---|---|---|---|
| Sham | saline | $0.89 \pm 0.23^{**}$ | $44.6 \pm 3.4^{**}$ | $50.2 \pm 12.4^{*}$ |
| Isc-Rep | saline | $1.50 \pm 0.24$ | $37.4 \pm 5.8$ | $38.5 \pm 6.9$ |
| Copper aspirinate | 7.5 | $1.10 \pm 0.18^{**}$ | $55.2 \pm 7.9^{**}$ | $40.5 \pm 6.6$ |
| | 15.0 | $1.02 \pm 0.16^{**}$ | $59.0 \pm 9.9^{**}$ | $44.3 \pm 7.1$ |
| | 30.0 | $0.82 \pm 0.23^{**}$ | $63.3 \pm 13.4^{**}$ | $45.8 \pm 7.2$ |
| Aspirin | 30.0 | $0.96 \pm 0.28^{**}$ | $40.9 \pm 7.5$ | $44.7 \pm 7.2$ |

Values were means ± s.d, $n = 6$. $^{*}P < 0.05$, $^{**}P < 0.01$ compared with Isc-Rep group by Student's *t*-test.

During reperfusion, a significant increase of MDA content in the cortex was observed in the Isc-Rep group as compared with the sham-operated group ($P < 0.01$). All drugs treated in this study significantly inhibited the increase of MDA contents (Table II). In the Isc-Rep group, the activities of SOD and GSH-PX significantly reduced after ischemia-reperfusion injury ($P < 0.05$ compared with the sham-operated group). Copper aspirinate (7.5, 15.0, and 30.0 mg $kg^{-1}$) significantly enhanced SOD activity, while aspirin even at 30 mg $kg^{-1}$ did not influence the SOD activity. The effect on GSH-PX activity was not observed in all the drug-treated groups (Table II).

Reactive oxygen species have been implicated to play a central role in neuronal damage. The brain is especially sensitive to oxygen species-induced lipid peroxidation due to the abundant polyunsaturated fatty acids and the low antioxidant enzymatic activities in the brain [10]. It has been demonstrated that SOD and GSH-PX, the major endogenous antioxidants in the brain, are the most important enzymes for neuronal defense against peroxide toxicity *in vitro* [11]. The present results shows that copper aspirinate given intragastrically elevates the SOD activity in a dose-dependent manner during reperfusion and that aspirin does not. Obviously, the anti-peroxidation mechanism of copper aspirinate is different from that of aspirin. Copper aspirinate mainly increases the activity of SOD to attenuate lipid peroxidative damage.

In conclusion, copper aspirinate has the potential to become a useful neuroprotective agent. Its protection may be attributed to many pharmacological properties, such as increasing SOD activity, anti-calcium overloading, inhibiting platelet aggregation and adhesion as well as increasing the prostacyclin level.

### REFERENCES

1. Shen, Z.Q., Lei W.Y., Li, L., Chen, Z.H., Liu, W.P. *Inflammopharmacology*, 1998, **6**, 357-362
2. Shen, Z.Q., Liang, Y., Chen, Z.H., Liu, W.P., Duan, L. *J. Pharm. Pharmacol.*, 1998, **50**, 1275-1279
3. Li, L., Wu, L.O., Liu, W.P., Xiong, H.Z., Yang, Y.K., Chen, Z.H. *Acad. J. Kunming Med. Coll.*, 1996, **17**, 1-3
4. Liu, W.P., Li, L., Xiong, H.Z., Shen, Z.Q., Yang, Y.K., Chen, Z.H. *Metal-based Drugs*, 1998, **5**, 123-126
5. Bradford, M.M. *Anal biochem*, 1976, **72**, 248-254
6. Ohkawa, H., Ohishi, N., Yagi, K. *Anal Biochem*, 1979, **95**, 351-358
7. Shen, Z.Q., Li, L., Wu, L.O., Liu, W.P., Chen, Z.H. *Platelets*, 1999, **10**, 345-348
8. Yang, W.M., Shen, Z.Q., Chen, Z.H., Li, L., Peng, F., Liu, W.P. *Acta Pharmacol. Sin.*, 2001, **22**: 121-124

9. Shen Z.Q., Wu L.O., Liu W.P., Liu J.K., Chen Z.H. *Metal-based Drugs*, 2001, **8**, 103-105
10. Dargel R. *Exp Toxicol Pathol*, 1992, **44**, 1169-1181
11. Desagher, S., Glowinski, J., Premont, J. *J. Neurosci.*, 1996, **16**, 2553-2562

**Received: January 28, 2002 – Accepted: January 30, 2002 – Accepted in publishable format: January 31, 2002**

